# "We can move forward": challenging historical inequity in public health research in Solomon Islands

**DOI:** 10.1186/1475-9276-9-25

**Published:** 2010-11-05

**Authors:** Michelle L Redman-MacLaren, David J MacLaren, Rowena Asugeni, Chillion E Fa'anuabae, Humpress Harrington, Alwin Muse, Richard Speare, Alan R Clough

**Affiliations:** 1 School of Public Health, Tropical Medicine and Rehabilitation Sciences, James Cook University, McGregor Road, Smithfield, Cairns, Queensland, Australia; 2 Atoifi Adventist Hospital, Uru Harbour, East Kwaio, Malaita Province, Solomon Islands; 3 Atoifi College of Nursing, Uru Harbour, East Kwaio, Malaita Province, Solomon Islands; 4 School of Public Health, Tropical Medicine and Rehabilitation Sciences, James Cook University, James Cook Drive, Douglas, Townsville, Queensland, Australia; 5 School of Indigenous Australian Studies, James Cook University, McGregor Road, Smithfield, Cairns, Queensland, Australia

## Abstract

**Background:**

In resource-poor countries, such as Solomon Islands, the research agenda on health is often dominated by researchers from resource-rich countries. New strategies are needed to empower local researchers to set directions for health research. This paper presents a process which seeks to enable a local and potentially more equitable research agenda at a remote hospital in Solomon Islands.

**Methods:**

In preparation for a health research capacity-building workshop at Atoifi Adventist Hospital, Malaita, Solomon Islands, a computer-based search was conducted of Solomon Islands public health literature. Using a levels-of-agreement approach publications were categorised as: a) original research, b) reviews, c) program descriptions and d) commentaries or discussion. Original research publications were further sub-categorised as: i) measurement, ii) descriptive research and iii) intervention studies. Results were reviewed with Solomon Islander health professionals in a focus group discussion during the health research workshop. Focus group participants were invited to discuss reactions to literature search results and how results might assist current or future local researchers to identify gaps in the published research literature and possible research opportunities at the hospital and surrounding communities. Focus group data were analysed using a grounded theory approach.

**Results:**

Of the 218 publications meeting inclusion criteria, 144 (66%) were categorised as 'original research', 42 (19%) as 'commentaries/discussion', 28 (13%) as 'descriptions of programs' and 4 (2%) as 'reviews'. Agreement between three authors' (MRM, DM, AC) independent categorisation was 'excellent' (0.8 <*κ*). The 144 'original research' publications included 115 (80%) 'descriptive studies' (*κ *= 0.82); 19 (13%) 'intervention studies' (*κ *= 0.77); and 10 (7%) 'measurement studies'(*κ *= 0.80). Key themes identified in the focus group discussion challenged historical inequities evident from the literature review. These included: i) who has done/is doing research in Solomon Islands (largely non-Solomon Islanders); ii) when the research was done (research needs to keep up to date); iii) amount of published research (there should be more); iv) types of research (lack of intervention and operational research); v) value of published research (important); vi) gaps in published literature (need more research about nursing); vii) opportunities for research action (start small); viii) support required to undertake research at the hospital and in surrounding communities (mentoring and partnering with experienced researchers).

**Conclusions:**

A search and collaborative review of public health literature for Solomon Islands at a health research capacity building workshop has uncovered and challenged historical inequity in the conduct and access to public health research. Emerging Solomon Islander researchers at a remote hospital are now working to set priorities and strengthen local research efforts. These efforts have highlighted the importance of collaboration and mentoring for Solomon Islanders to instigate and implement public health research to improve the health of individuals and communities served by this remote hospital.

## Background

Health research can provide evidence to guide public health practice and contribute to our understanding of health issues. The public health profession faces a challenge to make its research generate improved health outcomes for research participants and their communities [1]. This challenge is particularly pertinent in a resource-poor country like Solomon Islands, to ensure that scarce research infrastructure and resources are allocated toward effective, targeted public health research for more equitable population health outcomes [2].

Internationally, little has been published about how public health research priorities are set, in particular how health professionals can directly influence the research agenda [3]. There are also limited publications in the area of intervention and operational research that test the effectiveness or performance of public health programs [1,4]. Public health research has the potential to be more equitable through collaborating with local health professionals and local community members. Involvement by those who deliver or inform public health programs and services is critical because of their intimate awareness of local health issues and needs [5].

This paper describes the process of supporting a local and potentially more equitable health research agenda at a remote hospital and college of nursing in Solomon Islands. The paper describes two stages: (i) reporting the amount and nature of public health research from/about Solomon Islands compiled in a literature search, and (ii) the exploration of responses to results of the literature search with a group of health professionals participating in a health research workshop.

### An Invitation

While attending a conference in Cairns, Australia in September 2008 senior staff from Atoifi Adventist Hospital (AAH), Atoifi College of Nursing (ACON) and an East Kwaio chief (who is also a health worker) were hosted by public health researchers (DM and AC) from James Cook University (JCU). All of these senior health workers from Solomon Islands had a long-standing relationship with DM, who has engaged in health service delivery and public health research in East Kwaio since 1992. During a discussion about the need for health research to inform policy and practice at AAH these leaders requested that JCU facilitate an 'Introduction to Health Research' workshop at AAH. The workshop would aim to assist in strengthening capacity of staff and students in health research while exploring collaborative research opportunities.

### Solomon Islands

Solomon Islands is a nation of over nine hundred islands, from high mountainous ones to low-lying coral atolls. The country gained independence from Great Britain in 1978. More than 80% of the population of 595 000 live a village-based subsistence lifestyle, speaking over 70 indigenous languages [6,7]. Solomon Islands is re-establishing civil and political systems damaged by ethnic conflict between 1998-2003, locally referred to as the 'Tension' [8,9]. The country's health system is heavily donor-reliant. Recent health system initiatives include a sector-wide approach to health, which emphasises a Ministry of Health-mandated strengthening of health sector management and greater co-ordination of health services with the national health plan [10-12]. However, the health system has to date failed to meet many identified health targets [13]. The 2008/09 global financial crisis reduced resources available for health services across the country, seriously impacting health services for Solomon Islanders (personal communication E. Riberyo, 3 July 2009).

### Atoifi Adventist Hospital and Atoifi College of Nursing

Atoifi Adventist Hospital (AAH) is a 90-bed general hospital established in 1966 in East Kwaio on the remote east coast of the island of Malaita. It is the largest non-government hospital in Solomon Islands and operates the only college of nursing outside the nation's capital city. It provides direct medical and surgical services to over ten thousand villagers who live on the coastal fringes and mountainous interior of the island [14]. Patients often travel from across Malaita or from other provinces to seek medical services there. Access to AAH is by a twice-weekly light aircraft to a grass airstrip, irregular shipping service or motorised canoe. There are no roads to the hospital, and local villagers must walk rainforest trails to get there, sometimes for many hours. Most staff and students live on campus. All power generation, water supply, communication infrastructure, building construction and maintenance are managed by AAH. The hospital also manages a store, bank agency and airline agency. The only public telephone in East Malaita (population approx 50,000) is located on the hospital campus. Limited infrastructure and financial constraints are exacerbated by the remote location. At the time of writing, electricity is available for two four-hour periods per day, which limits telephone and internet access. Limited internet bandwidth also means online access to health literature is slow and difficult. Access to online health literature is available via the World Health Organisation (WHO) HINARI health database, although infrastructure constraints prevent routine AAH usage.

Atoifi College of Nursing (ACON) is one of two Colleges of Nursing in Solomon Islands. It is located on the AAH campus with up to 60 students pursuing a three-year Diploma of Nursing. Many ACON graduates provide clinical nursing care in provincial or national hospitals, manage rural clinics or administer provincial/national public health programs. Major population health issues at AAH include: high rates of malaria, tuberculosis, respiratory and skin infections, childhood malnutrition, diabetes, anaemia in pregnancy and parasite infestation.

Research capacity at the hospital and college of nursing is limited. There has been some heath research undertaken at the hospital and in surrounding areas [15-18], with authors DM, HH, RA having previously collaborated on health research projects at AAH [14,19,20]. AAH/ACON have expressed desire to increase research capacity and for Solomon Islanders to instigate and implement health research at AAH and surrounds to enable a more local and equitable research agenda.

## Methods

### Literature Search

#### Search strategies

Adapting established approaches used by Gilligan et al. [21] and Sanson-Fisher et al. [1,22] MRM, AC and DM searched for and classified primary peer-reviewed literature. The databases *PubMed*, *PsycINFO *and *Scopus *were interrogated. *PubMed *includes all Medline entries for peer-reviewed health and medical journals. *PsycINFO *includes 99% peer-reviewed material from a variety of behavioural and social science journals. *Scopus *is a large database which interrogates almost 18,000 peer-reviewed online journals in areas of science, life sciences, physical sciences, medicine and social sciences. These databases have a strong focus on health, public health and health sciences[23-25].

The search term "Solomon Islands" was used. Studies were initially reviewed if there was a reference to "Solomon Islands" in the title, abstract or article text. Only studies with a population health focus were included. Excluded were studies that were primarily genetics research or reports of laboratory-based virological investigations, or did not directly involve human populations. Also excluded were publications which referred to Solomon Islands but were not primarily about Solomon Islands, or were not based on research undertaken there. Articles or key information about articles was imported into and managed using *Endnote *(Version X2.0.1).

#### Classification of literature

Adapting the methodology and definitions of Sanson-Fisher et al. [1], literature was classified into four categories: a) original research, b) reviews (both systematic and critical), c) description of programs and d) commentaries or discussion. In the original research category, three sub-categories were identified: i) measurement studies, ii) descriptive studies and iii) intervention studies.

#### Quantitative Data analysis

MRM, AC and DM independently categorised the literature retrieved. The kappa statistic was used to assess the level of agreement between the three authors (Stata 9). Agreement was assessed using six categories of publication, and also using four categories with the three different sub-categories of the 'original research' category combined into one group.

For the final categorisation, articles were assigned the category agreed upon by two or more of the authors. For the 12 articles in results where there was no initial agreement, that is where there were three different categories initially assigned to an article, two of the authors (MRM, AC) further reviewed the articles and made a determination by consensus. The number of publications in each five-year period was examined to assess changes over time.

The literature search, classification and quantitative analysis were undertaken at JCU (Australia) during June - August 2009 in preparation for presentation and discussion at the September 2009 research workshop. It would have been preferable, and more consistent with participatory action research methodology to have undertaken the literature search and associated analysis at AAH. This was not possible due to the logistical and infrastructure constrains as described above.

#### Workshop methodology

The five-day 'Introduction to Health Research' workshop facilitated at AAH/ACON in September 2009 covered basic research concepts and methods with health professionals, teachers, community leaders, chiefs and auxiliary hospital workers. The workshop was co-designed, evaluated and reported upon by hospital, college and JCU staff (unpublished report). A participatory action research approach [5,26] informed the development and facilitation of the workshop following the definition by Stringer & Genat: *"a systematic, participatory approach to enquiry that enables people to extend their understanding of problems and issues and to formulate actions directed towards the resolution of these problems and issues" *[5]. Critical decolonising research methodologies also underpinned the workshop which ensured knowledge from participants and facilitators were exchanged and critiqued to develop research skills appropriate for the hospital and surrounding communities [27-29]. Emergent issues were continuously identified and responded to throughout the workshop, ensuring responses were grounded in the local context.

#### Focus Group Discussion

On day two of the workshop, a focus group discussion [30] was facilitated by MRM and notes recorded by DM. The focus group members were invited to:

1. Discuss reactions to results of the literature search

2. Discuss how results from the literature search might assist current or future researchers

3. Identify gaps in the published research literature

4. Identify possible research opportunities, including types of research and specific research topics

#### Qualitative Data Analysis

Focus group data were analysed using a grounded theory approach [31,32]. Units of meaning, emergent codes and themes were identified by MRM. RA, CF and HH reviewed and further developed the analysis with MRM in December 2009. Data analysis was then finalised and presented to staff and students at the hospital and college.

## Results

### Literature Search

Articles identified using the search term "Solomon Islands" were imported from PubMed (349), PsycINFO (29) and Scopus (106). After removing duplications and applying exclusionary criteria, 218 articles remained to be evaluated. The numbers in each category were: 144 (66%) original research; 42 (19%) commentaries/discussion papers; 28 (13%) description of programs; and 4 (2%) reviews. The numbers in the three sub-categories of original research were: 115 (80%) descriptive studies; 10 (7%) measurement studies and 19 (13%) intervention studies.

Agreement between the authors' (MRM, AC, DM) independent categorisation was 'good' (0.6 <*κ *< 0.8) [33] when the 218 items were classified into the six categories, but rose to 'excellent' (0.8 <*κ*) [33] when the three subcategories of 'research' were assessed as one category. Final agreement was 'good' to 'excellent' when MRM, AC and DM categorised the 144 original research papers as either: 'intervention' (*κ *= 0.77), 'measurement' (*κ *= 0.80) or 'descriptive' (*κ *= 0.82).

**Figure 1:**
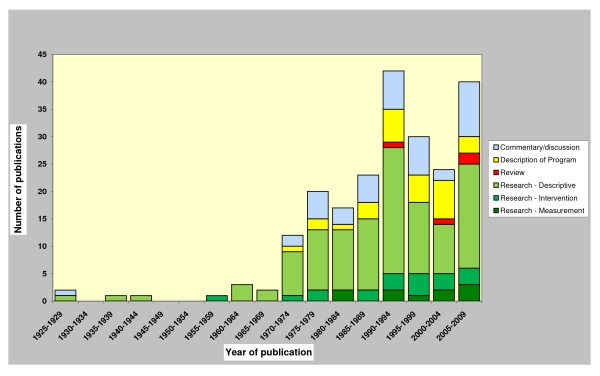
**Public Health Literature Solomon Islands 1928-2009**.

The number and type of publications are presented in five-year blocks from 1928-2009 in Figure 1. The number of articles about Solomon Islands has generally increased over time. Two five-year periods show significantly more research activity: 1990-1994 and 2005-2009. The decrease in research publications between 1995 and 2004 is no surprise given the breakdown between 1998-2003 of government systems, including law and order that resulted in civil unrest. Much literature is devoted to the description and treatment of particular diseases. This included between 1990 and 2009, 26% (35/137) of the publications were on malaria, 12% (17/137) on sexually transmitted diseases including HIV/AIDS, 5% (7/137) on mental health and 2% (3/137) on tuberculosis (data not shown). Nursing in Solomon Islands was mentioned in the title or abstract in 2% (5/218) of total publications, although only two publications were found to focus specifically on nursing practice/education. One was about distance learning models for nursing education[34] and one about the role of nursing in malaria prevention and treatment [35].

A small number of the authors contributed multiple publications to the Solomon Islands literature, including: Eason 12/218 with a number of clinical case studies (1994-1989); Kere on malaria 8/218 (1987-1996) and Mazzur on hepatitis 6/218 (1976-1981). Solomon Islander authors have generally been poorly represented in public health literature. Solomon Islander authors of this paper reviewed publications between 1999 and 2009 and identified 16/70 (22.9%) publications to have Solomon Islanders as lead author.

### Workshop Results

The 'Introduction to Health Research' workshop was held at AAH/ACON over five days in September 2009, with 102 participants. JCU health researchers facilitated the workshop at the invitation of AAH/ACON leaders, assisted by emerging health researchers from the hospital, college of nursing and a community chief. Two sessions were held daily: a morning session predominantly conducted in Solomon Islands Pijin and an evening session predominantly conducted in English. Following a workshop session facilitated by MRM on conducting a literature search for health research, workshop participants were invited to participate in a focus group to discuss the results of the literature search. Four workshop participants voluntarily joined the focus group discussion to review and analyse the literature search. The four participants all hold senior positions at AAH or ACON but came from different provinces across Solomon Islands: Malaita Province, Western Province, Makira Province. The Malaitan author (CE) is from East Kwaio, where the hospital is located. This diverse range of focus group participants, although small in number, allowed for focus on both the immediate East Kwaio context and more broadly in Solomon Islands. All four focus group members are authors of this paper (RA, HH, AM, CE). The group was a naturally occurring focus group and used both Solomon Islands Pijin and English [36]. The shared authorship between AAH/ACON and JCU is consistent with action research methodologies which seek to diminish the distinction between 'researched' and 'researcher', and allows for those experiencing the problems or issues to examine the available information and together work on possible solutions.

### Focus Group Discussion

#### Focus Group Themes

Table 1 shows comments, emerging codes and themes from the focus group discussion. A summary follows.

**Table 1:** Themes, Codes and Participant Comments from Focus Group Discussion

Emergent Themes	Codes emerging from focus group data	Focus Group Discussion participant comments
Responses to results of the Literature Search	Who is doing public health research in Solomon Islands	*We need to do more research ourselves* *and not (shouldn't be) from outside* *Outside people [are] doing research* *Need to have more Solomon Islanders so have the best interests of the country*
	When was the research done	*A lot of research done many years back and there is more needed to keep up to date. We need to keep up and have current knowledge* *The research in 1980s we need to look at this but also current*
	Amount of research	*There should be more studies* *There are lots of things to be surveyed*
	Types of research	*There is more on description* *We need to move on invitation to make changes* *When look at Solomons- need to have current research on interventions* *Good to have description*
	Value of research	*Looking at literature is important* *Need to look at this and learn from it*
	Gaps in literature	*There are not many articles about nursing practice in Solomon Islands* *This is important given the role of nursing in SI*
Opportunities for research action	Identified Opportunities	*To look at approaches and have real data* *Look at approaches to decreasing diseases* *We need to increase research* *Have not had expectations to do publications but now looking at doing this* *Want to do things about nursing practice and management* *Solomon Islands need to have a good understanding of research* *Need to start on small research and then more questions will come and we will learn from this. This research will work as a catalyst for more* *Need simple area (of research)* *You need to be clear about your area of research*
Moving Forward: Mentoring and Support	Research support needs	*Many are new to research* *How to look at research is difficult* *Some of us want to do it (research) but have no knowledge of how to write articles*. *We need help to increase research practice* *Some people have done research but not published it* *We need mentors and continuous support to do good research from this and have good results* *After this we might be frustrated and not have anyone to support us*

#### Theme One: Responses to results of the literature search

The literature was reviewed by AAH/ACON focus group participants in light of the literature search undertaken by JCU authors. The issue of who has conducted public health research in Solomon Islands stimulated considerable discussion: *"We need to do more research ourselves"; "not outside people doing research"*. The periods when particular research was undertaken and the need to stay up to date with current findings were highlighted: *"[we need to] keep up and have current knowledge", "research in the 1980s: need to look at this but also [be] current"*. It was opined that there *"should be more studies" *since there are *"lots of things to be surveyed"*. The value of research and the gaps in the literature topic included: *"looking at the literature is important" *and *"there are not too many articles about nursing practice in Solomon Islands" *(2/218).

#### Theme Two: Opportunities for research action

It became obvious the literature search could influence the nature and foci of future health research at the hospital. During the focus group discussion the view emerged that there was considerable opportunity to undertake research which could improve the health of local populations and make a contribution to reducing disease. Focus group discussion participants were surprised by the small number of articles in the literature search, noting: *"there should be more studies"*; *"we need to move on invitation to make changes"*. Focus group discussion participants were particularly surprised at the dearth of publications about nursing practice, education and management in Solomon Islands (1/218) given the central role nurses play in the planning, provision and evaluation of health services in Solomon Islands.

The importance of using research as a catalyst for positive changes to health also emerged from focus group discussion. An opportunity to *"start on small research" *and then to *"increase research" *was identified. *"More questions will come and we will learn from this"*. Also highlighted was the importance of operational research as a means of advancing health interventions such as health education and health promotion. An example of operational research, planned by one author, is to investigate the impact of weekly public health education presentations given to patients and their families by hospital staff.

Definitions

**Intervention Study: **Tests the effectiveness of an intervention to modify preventative health-risk behaviours and/or the implementation of best practices by healthcare professionals [1]

**Operational Research (OR): **Provides decision-makers with information to enable them to improve the performance of their programs. Operational research helps to identify solutions to problems that limit program quality, efficiency and effectiveness, or to determine which alternative service delivery strategy would yield the best outcomes [4]

#### Theme Three: Moving forward--mentoring and support

Ongoing support to progress the research agenda at AAH/ACON was identified as essential: *"Some of us want to do it (research) but have no knowledge of how to write articles"*.

Mentoring of researchers at the hospital and college was identified as vital as *"many of us are new to research". It was reported that "We need mentors and continuous support to do good research from this and have good results" *otherwise *"we might become frustrated and not have anyone to support us." *Such support from experienced researchers and institutions will assist, because *"we need help to increase research practice"*.

## Discussion

### The Literature Search

The importance of reviews of public health literature is well documented [1,21,22]. This public health literature search is distinctive not as a specified sub-group or public health issue, but because of its geographic focus on an entire country. The review and application of the literature search with emerging researchers at AAH/ACON supported by researchers from JCU, offered a more local and equitable process of examining the published public health literature. It also contributed to raising consciousness and further supported the strengthening of health research at this remote Solomon Islands hospital and college of nursing.

### Challenging Historical Inequities

The collaborative application of the literature search at AAH/ACON provided an avenue to raise consciousness about the types of research that have been undertaken in Solomon Islands and potential opportunities for increasing research activity by Solomon Islanders at AAH/ACON. The need for a move to research action, including research that will result in improved community health outcomes emerged in focus group discussion. Although the predominant form of research in Solomon Islands has been descriptive research, and though descriptive studies do not necessarily lead directly to better health outcomes [1,37], the focus group discussion emphasised the importance of descriptive studies since there are still *"lots of things to be surveyed*". Because published studies are few, and because there are many complex public health issues, all types of research need to be strengthened to expand the evidence base for Solomon Islands. The research workshop, including the focus group discussion process and subsequent reflection, has provided an opportunity to consider how health research priorities should be set at AAH/ACON [38,39]. Systems are now being planned and implemented at the hospital and college to set research priorities and support research activity. A research committee has since been established and the AAH ethics committee processes reviewed. As expressed by one focus group participant: "*We can move forward*".

Because emerging researchers at AAH/ACON are directly involved in health service delivery or nursing education, they are intimately aware of the health issues which require further research. This allows collaborative research priorities to be developed in partnership with other health service providers and research institutions [3]. This is important for intervention and operational research because it represents potential for health professionals who plan and implement health programs to be central to research priority setting and activity. This is particularly important for a hospital and college of nursing such as AAH/ACON located in a remote location and who serve a rural village population.

Health professionals and emerging researchers from AAH/ACON and JCU have in partnership examined public health research and explored possibilities of future public health research--a step towards re-imagining the externally driven, inequitable, predominantly Westernised research process as it has been experienced by many Solomon Islanders. The collaborative review and application of the literature search has the potential to influence the national health research agenda with many AAH/ACON staff also members of national hospital, public health and tertiary education boards. Since the workshop, staff of the Solomon Islands Medical, Training and Research Institute (SIMTRI) have reported that the AAH workshop has stimulated an interest in strengthening research capacity of Solomon Islanders at the national level.

The consequence of presenting the results of the literature search at the research workshop and collaboratively reviewing and analysing the literature search in focus group discussion with emerging Solomon Islander researchers enabled it to be grounded in the reality of a remote Solomon Islands hospital context. The process highlighted many of the constraints present while planning and conducting public health research in locations with resource, infrastructure and logistical constraints. However, the partnership between the local (indigenous) and external (Western) contexts (AAH and JCU) has demonstrated the potential of such processes to build more equitable research agendas in similar remote or resource challenged contexts.

### Limitations

Ideally, we would have preferred that the literature search would have been undertaken at AAH/ACON with all authors directly involved rather than being undertaken at JCU in Australia, and quantitatively analysed prior to the workshop in Solomon Islands. However, this was not possible due to limited resources and infrastructure at the hospital and college and the need for the literature search to be compiled prior to the workshop. This raises ethical issues about equitable access to information and the construction of health research discourses. One small step to address the difficulty in accessing information has been the Anton Breinl Centre for Public Health and Tropical Medicine at JCU to establish JCU adjunct researcher positions for emerging researchers at AAH/ACON. This is facilitating access to online resources, important computer programs and exchange between personnel at JCU and AAH/ACON. It is also providing a point of contact to facilitate ongoing research collaboration between AAH/ACON and JCU. Knowledge of how literature searches can be conducted and analysed in a remote location has been exchanged with this expanding the possibilities for evidence-based research practice at the hospital and college of nursing.

A further limitation was the small number of focus group participants. Many workshop participants said that concepts such as literature searches, publications, journals and articles were new to them. Some said that being invited to a focus group discussion to discuss and analyse a literature search early in the workshop had been overwhelming. Lack of follow up by some training facilitators at the hospital in the past also left some workshop participants doubtful as to the value of participating in the focus group discussion. Although this was unfortunate, the members of the focus group discussion were all senior hospital or college staff with the ability to strengthen and support the health research agenda at the hospital and surrounding communities. Two of the focus group participants (RA and HH) have subsequently travelled to JCU where plans progressed for further research at the hospital and surrounding communities.

Because time was limited, only one focus group discussion was facilitated, and this limited conceptual saturation [32]. However, all four focus group participants analysed the focus group discussion data in a manner consistent with participatory action research methodologies, and all are authors of this paper. MRM returned to AAH/ACON seven weeks after the workshop and worked directly with authors to review the focus group discussion data analysis. RA, CF, HH and MRM collaboratively reported the focus group discussion results to AAH/ACON staff and students during a weekly professional development meeting.

## Conclusions

The search, analysis and collaborative review of Solomon Islands public health literature has enhanced health research equity by being a valuable contribution to both established and emerging public health researchers and health practitioners. A focus group discussion about the literature search with a group of health professionals at the hospital and College of Nursing has shown a divergence between the amount and type of research that has been undertaken to date and the needs of a remote hospital such as AAH. AAH/ACON researchers have identified a particular gap in the area of nursing practice, education and management in Solomon Islands.

The application of the literature search at AAH/ACON has influenced the nature and focus of future health research at this remote Solomon Islands institution by stimulating research interest, identifying gaps in health research and highlighting the need for Solomon Islanders to undertake research. It has also helped to highlight the importance of research being able to influence health outcomes for the rural majority in Solomon Islands. As a part of the health research capacity building workshop, the collaborative review of the literature search has contributed to further developing the knowledge and enthusiasm of Solomon Islander researchers to undertake heath research in the populations where they live and to which they belong. AAH/ACON has requested that JCU provide further support and mentoring to develop local research capacity that can influence and help inform research at the national level through collaboration with the Ministry of Health and Medical Services (MHMS), Solomon Islands Institute of Medical, Training and Research Institute (SIMTRI) and other partners.

It is time to support and strengthen the capacity of Solomon Islander researchers to plan, undertake and report upon public health research. This is, of course, also the case in similar resource-poor countries. In undertaking a search and collaborative review of public health literature from Solomon Islands during a health research capacity building workshop and then collaboratively publishing the resultant possibilities, a new approach is being forged at AAH/ACON. AAH/ACON and JCU researchers uncovering possibilities for health research in Eastern Malaita are initial steps in a journey to improve individual and population health for local, provincial and national populations. The approach also contributes to a clearer, more equitable and informed health research agenda at this remote hospital and college of nursing. It begins to respond to the challenge put to public health researchers to undertake research that improves health outcomes for individuals and their communities.

## Competing interests

The authors declare that they have no competing interests.

## Authors' contributions

MRM undertook the literature search, facilitated the focus group discussion, led the focus group discussion data analysis, drafted and edited the manuscript. DM recorded focus group discussion data, contributed to the analysis of literature search and focus group discussion data and edited the manuscript. RA, CF, HH and AM participated in focus group discussion, analysed the focus group discussion data and contributed to the manuscript. RS co-facilitated the design and coordination of the study and contributed to the manuscript. AC led the design and coordination of the research, performed the statistical analysis and edited the manuscript. All authors read and approved the final manuscript.
